# Alexithymia and Emotion Regulation Strategies in Adolescent Gamblers with and Without At-Risk Profiles

**DOI:** 10.1007/s10899-021-10057-8

**Published:** 2021-07-11

**Authors:** Ana Estévez, Paula Jauregui, Laura Macía, Cristina Martín-Pérez

**Affiliations:** Faculty of Psychology and Education, University of Deusto, Apartado 1, 48080 Bilbao, Spain

**Keywords:** Alexithymia, Emotion regulation, Affect, Pathological gambling, Adolescents

## Abstract

Alexithymia, difficulties in emotion regulation, and negative affect play an important role in adolescents who present pathological gambling. Therefore, the objectives of the present study were, firstly, to analyze the differences between alexithymia, difficulties in emotion regulation, and positive and negative affect in adolescents with and without risk of gambling problems. Secondly, the relationships between all the variables of the study in adolescents with and without risk of problem gambling were analyzed separately. Thirdly, we analyzed the mediating role of positive and negative affect in the relationship between alexithymia and dysfunctional emotion regulation strategies (ERS) in adolescents at risk of gambling problems. The sample was composed of 206 adolescents with ages ranging from 12 to 18 years (*M* = 15.52; *SD* = 1.43). They were divided into two groups according to the score obtained in the South Oaks Gambling Screen-Revised for Adolescents (SOGS-RA). Thus, 84 were included in the group without risk of gambling problems and 122 in the group at risk of gambling problems. The results obtained revealed higher scores in negative affect and pathological gambling in the group at risk of gambling problems. Likewise, positive relationships between alexithymia, maladaptive emotion regulation strategies (MERS), and affect were found. Mediation analyses showed that difficulties in identifying feelings were indirectly related to greater use of dysfunctional ERS through their relationship with negative affect in at-risk gamblers.

## Introduction

Gambling disorder, which has been included as an addictive disorder in the last DSM edition (APA, 2013), consists of persistent and recurrent gambling behavior despite its negative consequences. Gambling has become one of the most frequent addictive behaviors in adolescents and young adults (Secades-Villa et al., 2016). This may be due to the growing availability and accessibility of gambling, which may result in an increasing prevalence of gambling problems (González-Roz et al., 2017; Secades-Villa et al., 2014). A systematic review made by Calado et al. (2017) shows that 0.2%–12.3% of adolescents fulfill the criteria for problem gambling. It is known that many adults who present a gambling disorder start their risky gambling behavior during adolescence (Garrido et al., 2017; Sharman et al., 2019; Volberg et al., 2010). Moreover, an early start in gambling behavior is associated with greater severity in later stages of life (Jiménez-Murcia et al., 2010; Kang et al., 2019; Lamas et al., 2018).

Emotion regulation has been shown to be a central transdiagnostic factor associated with the etiology and maintenance of several psychological and addictive disorders, including gambling disorder (Jauregui et al., 2016; Sancho et al., 2019). Emotion regulation refers to a group of processes through which individuals influence and control their emotional states (McRae & Gross, 2020), allowing them to detect which emotions they feel, when they feel them, and how they experience and express them (Gross, 2002). In this sense, Shead et al. (2008) highlight that gambling disorder is directly related to the expectation of altering one’s mood, that is, alleviating negative affective states and achieving positive affect. Khantzian (1985) also formulated the self-medication theory, which holds that addictive behaviors are a means for alleviating negative affective states. In fact, Orlowski et al. (2019) found that a lack of the capacity for tolerating negative emotions is an important risk factor for developing problematic and pathological gambling behaviors, whereas non-problem gamblers showed greater use of acceptance strategies. In the same vein, authors such as Jauregui et al. (2016) observed that pathological gamblers showed greater emotion regulation difficulties in comparison with non-gamblers. Difficulties in emotion regulation predict gambling behavior and its severity (Rogier y Velotti, 2018). There are fewer studies conducted with adolescents, but they show similar results (Estévez et al., 2020; Marchica et al., 2019). Jara-Rizzo et al. (2019) also emphasize the importance of emotion regulation processes in the cognitive and behavioral manifestations of gambling behavior.

In the same line, authors such as Sheppes et al. (2015) have attempted to conceptualize the emotion regulation processes that underlie the gambling disorder, considering this process as the result of three main stages, starting with identification. This stage consists of the capacity of adequately representing an emotional state and rating the need for regulating it or not. Additionally, Rogier and Velotti (2018) suggest that individuals with problem-gambling show potential failures in the identification stage due to a deficit in emotional awareness, difficulties in accepting affective states, and personality traits such as alexithymia. Alexithymia is characterized by a scarce capacity of analyzing, describing, identifying, and differentiating one’s own and others’ emotional states (Brewer et al., 2016; Kandri et al., 2014). In this sense, Di Trani et al. (2017) point out that pathological gambling behaviors may act as external regulators of undifferentiated inner emotional states. Concurrently, although few studies analyze the relationship between alexithymia and gambling in adolescence, previous evidence shows that alexithymic traits may be associated with problematic gambling behavior and sensation-seeking, as well as with an increase in the severity of gambling symptoms, both in clinical and community samples (Marchetti et al., 2019; Morie et al., 2016; Sideli et al., 2018; Terrone et al., 2018). Moreover, Elmas et al. (2017) found that both alexithymia and difficulties in emotion regulation predicted gambling disorder and that individuals with higher scores in alexithymia may have a greater tendency to present addictive behavior through emotion dysregulation processes.

Likewise, recent studies conducted by Lyvers et al. (2017) found that expectations of regulating negative emotional states fully mediated the relationship between alexithymia and negative emotions, which may also be associated with deficits in emotion regulation. Moreover, Bradizza et al. (2018) highlighted that individuals who report greater alcohol abuse showed greater emotion regulation difficulties, had a greater tendency to use addictive behavior as a response to negative emotional states, and showed less attention and awareness to their own emotions. There is less research on gambling, but existing evidence indicates that gambling may also be related to the avoidance of negative emotional states and to maintaining positive emotional states (Blain et al., 2015). Nevertheless, negative urgency may play an important role in externalizing disorders such as gambling disorder (Johnson et al., 2017) and may be one of the strongest indicators of the psychopathological state among gamblers (Billieux et al., 2012). In the same line, Rogier et al. (2018) observed that gambling severity is associated with emotional suppression, which may also be related to negative urgency. Recent studies also reveal the neurobiological roots of negative urgency in basic processes of emotion regulation (Chester et al., 2016; Ruiz de Lara et al., 2018).

Despite the apparent relationship of the mentioned variables, there are few studies to date that have studied them concurrently in adolescent gamblers. Moreover, previous studies show that the relationship between gambling disorder and emotion regulation may be different in different samples of adolescents and young adults. Some studies have shown that gambling severity and emotion regulation may be related in clinical samples of individuals diagnosed with gambling disorder but not in community samples, whereas other studies do find this relationship in this type of samples (Estévez et al., , 2014, 2020). Therefore, more research is needed to clarify the role of emotion regulation as a function of the gambling profile (Jara-Rizzo et al., 2019). In the case of alexithymia, there is also a lack of studies analyzing different types of gambling profiles.

Therefore, the aims of this study were, first, to explore the differences among alexithymia, maladaptive emotion regulation strategies (MERS), and positive and negative affect among gamblers with and without problematic gambling behavior; second, to analyze the relationship among alexithymia, MERS, and positive and negative affect separately between both groups; and third, to study the mediating role of positive and negative affect in the relationship between alexithymia and MERS.

## Method

### Participants

A total sample of 206 adolescents (32% female) with an age range of 12–18 (*M* = 15.52, *SD* = 1.43) participated in this study. The sample was comprised of 96.5% of students, 2% of workers, and 1.5% who both studied and worked. Regarding the students, 41.5% were primary school students, and 49% were high school students, and the rest were university or professional career students (8.5%) or school drop-outs (1%).

This sample was divided into two groups (with and without at-risk gambling profiles).The group of gamblers without at-risk gambling profile (84 participants) comprised a 97.4% of students, a 1.3% of workers, and a 1.3% who were both workers and students. Among the students, 46.1% and 47.4%, respectively, were high school and primary school students, and only 1.3% and 5.1% were university and professional career students.

The group of participants who were at probable risk of gambling activity (122 participants) comprised a 96.5% of students, a 2.9% of workers, and a 0.9% of both students and workers. Among the students, 50.5% were high school students, 37.4% were primary school students, 9.6% were professional career students, 0.9% were university students, and 1.7% were drop-outs.

Those with a total score of zero in the SOGS-RA were excluded from this study´s analyses.

### Measures

#### Gambling Disorder

*The South Oaks Gambling Screen-Revised for Adolescents* **(**SOGS-RA; Winters et al., 1993), adapted to Spanish by Secades and Villa (1998), was used. The instrument is composed of 12 items that describe gambling behaviors during the last 12 months. All the items have dichotomous answers (yes/no), except for Item 1, which has four possible responses. The interpretation of the scores is as follows: 0–1 (non-problem gambling), 2–3 (at-risk gambler [ARG]), and 4 and above (problem gambling). The criteria used by the SOGS-RA to detect gambling problems are similar to the SOGS designed for adults (Lesieur & Blume, 1987), but the at-risk category combines current symptoms with symptoms that indicate the development of a subsequent gambling problem. The original instrument has adequate psychometric properties (Cronbach's alpha = 0.81). In the present study, Cronbach's alpha was 0.76.

#### Alexithymia

*The Toronto Alexithymia Scale-20* (TAS-20; Bagby et al., 1994a, b), adapted to Spanish by Martínez-Sánchez (1996), was used. This scale measures alexithymia through 20 items rated on a 6-point Likert response format ranging from 0 (*Strongly disagree*) to 5 (*Strongly agree*). The original scale is composed of three factors. Based on previous literature, in this study, we only used the DIF factor, which refers to problems identifying emotions and confusing them with physical symptoms. The global scale has shown good internal consistency (α = 0.83 for the original scale and α = 0.81 for the Spanish adaptation), and the DIF subscale had a Cronbach's alpha of 0.78. In the current study, Cronbach's alpha was 0.87 for the global scale, and 0.86 for the DIF subscale.

#### Emotion Regulation

*The Cognitive Emotion Regulation Questionnaire* (CERQ; Garnefski et al., 2001), adapted to Spanish by Domínguez-Sánchez et al. (2013), was used. The CERQ assesses the extent to which nine emotion regulation strategies (ERS) are employed to cope with negative life events. The questionnaire consists of 27 items rated on a 5-point Likert response ranging from 1 (*almost never*) to 5 (*almost always*). The nine strategies are grouped into two different factors. The first factor refers to emotional well-being and adaptive behaviors (i.e., putting into perspective, positive refocusing, positive reappraisal, acceptance, and refocusing on planning). The second factor includes maladaptive strategies associated with distress and psychopathological disorders (i.e., self-blame, other-blame, rumination, and catastrophizing). *Self-blame* refers to thoughts putting the blame for what you have experienced on yourself; (2) *other-blame* refers to thoughts of putting the blame for what you have experienced on the environment or another person; (3) *rumination* consists of obsessively focusing on the feelings and thoughts associated with a negative event; (4) *catastrophizing* refers to emphasizing and overestimating the negative experience or its consequences; (5) *putting into perspective* consists of thoughts brushing aside the seriousness of the event or considering its relativity when compared to other events; (6) *positive refocusing* refers to redirecting attention to joyful or pleasant themes; (7) *positive reappraisal* refers to reinterpreting the event in positive terms of personal growth; (8) *acceptance* includes thoughts of nonjudgmental resignation; and (9) *refocusing on planning* refers to thinking about the steps that should be taken to handle the situation resulting from the event. The original instrument presents adequate psychometric properties (α = 0.92 for the global scale, and α = 0.89 and 0.82 for the adaptive ERS and MERS subscales, respectively). In this study, Cronbach's alpha of the global scale was 0.84, while the respective subscales presented Cronbach's alphas of 0.73 and 0.81.

#### Positive and Negative Mood

*The Positive and Negative Affect Schedule* (PANAS; Watson et al., 1988), adapted to Spanish by Sandín et al. (1999), was used. The instrument consists of 20 words that describe emotions and feelings grouped into two categories: positive affect and negative affect. Each of these categories contains 10 items. Respondents indicate on a 5-point Likert response scale whether they are experiencing these emotions now or in the last two weeks (1 = *not at all/very slightly,* 5 = *very much*). Scores on each subscale range from 10 to 50 points, with higher scores indicating more affect. Both subscales show high internal consistency (α = 0.85 and 0.89, respectively, for Positive and Negative Affect). In the current study, the Positive and Negative Affect subscales showed alphas of 0.84 and 0.85, respectively.

### Procedure

Clinical and non-clinical samples were collected. The non-clinical sample was obtained through educational institutions of the Basque Country (northern Spain) and other nearby educational institutions, using convenience sampling.. An email was sent to the educational institutions that were likely to have adolescent and/or young adult students. In total, 545 school directors were contacted, of whom 18 directors responded and 10 agreed to participate. On the other hand, the clinical sample was recruited from gambling disorder treatment centers. The institutions participating in the study received feedback on the results of the research. This sample was divided into two groups (with and without at-risk gambling profiles) as a function of their total score on the SOGS-RA. The group of gamblers without at-risk gambling profile included those who had engaged in at least one gambling activity in the last year (a SOGS-RA score of 1). The second included those who were at probable risk of gambling activity (SOGS-RA score of 2 or more).

All participants were informed about the study and gave informed consent. Parental consent was requested for minors. Participation was voluntary, and confidentiality and anonymity were guaranteed. Participants did not receive any reward for collaborating in the study. Participants completed the questionnaire in online or offline versions. The questionnaire included general information about the main goals of the study, informing that there were no right or wrong answers and that participants could email the research team if they required further information about the study. The Institutional Review Board of the first author’s university approved the study.

### Statistical Analyses

#### Behavioral Measures and Mediation Analysis

To perform the parallel mediation analysis among our variables of interest, we first carried out Spearman's two-tailed correlations with SPSS. To complement the above analyses, we tested whether the relationship between higher difficulties to identify feelings (DIF; X) and higher use of MERS (Y) was mediated by negative (M1) or/and positive (M2) affect, using parallel mediation analysis (PROCESS macro v3.5 by Hayes) implemented in SPSS (v26; IBM, Chicago, IL, USA). This mediation analysis tested whether DIF predicted the negative/positive affect (path a_1_ and a_2_, respectively); whether negative/positive affect predicted MERS (path b_1_ and b_2_, respectively); and whether DIF predicted MERS (path c). In parallel mediation, two variables (M1 and M2) were proposed to mediate the association between X and Y; thus, two indirect effects (a_1_b_1_ and a_2_b_2_) will be explored. Next, this model tested the direct effects of DIF on MERS (path c’). Age and sex were included as covariates of no interest in the mediation analyses performed for both groups. A 95% bias-corrected confidence interval based on 5000 samples was calculated. In the ARG, we also controlled for the SOGS total score of the at-risk participants.

## Results

### Preliminary Analyses

The study groups were matched in age and sex. The clinical and sociodemographic characteristics of the sample are detailed in Table 1. Correlational analyses were also conducted between the variables of interest before testing our mediational hypotheses in each group separately (see Tables 2 and 3).

**Table 1:** Demographics and clinical characteristics of the groups

	At-risk problem gambling (*N* = 122) Mean (SD)	Non-problematic gambling (*N* = 84) Mean (SD)	Statistic^a^	*p* value
Age	15.68 (1.51)	15.29 (1.28)	1.948	.053
Sex (females)	41 (33.6%)	25 (29.8%)	.275	.600
Alexithymia-Difficulty identifying feelings (DIF)	17.05 (6.17)	16.24 (6.59)	1.043	.298
Positive affect	27.92 (7.81)	27.84 (7.00)	.074	.941
Negative affect	21.46 (7.02)	19.31 (6.55)	2.512	.013
Maladaptive ERS	20.26 (6.43)	19.15 (6.14)	1.236	.218
SOGS-RA (Total score)	4.24 (2.46)	1 (0)^b^	14.369	.000

^a^Independent samples t-tests were used to asses for between-groups differences in all cases, except for sex where chi-square tests were employed

^b^Non-problematic gambling group only included those with a total score of 1 in the SOGS questionnaire. *Abbreviations:* ERS, emotion regulation strategies

**Table 2:** Correlation matrix with data of the problematic gambling group (SOGS ≥ 2)

	1	2	3
1. Maladaptive ERS			
2. Alexithymia-Difficulty identifying feelings (DIF)	.330**		
3. Negative affect	.389**	292.**	
4. Positive affect	.235**	.170	.320**

**Correlation is significant at *p* < .01 (bilateral)

**Table 3:** Correlation matrix with data of the non-problematic gambling group (SOGS = 1)

	1	2	3
1. Maladaptive ERS			
2. Alexithymia-Difficulty identifying feelings (DIF)	.397**		
3. Negative affect	.243*	.377**	
4. Positive affect	.149	.041	.237*

**Correlation is significant at *p* < 0.01 (bilateral)

^*^Correlation is significant at *p* < 0.05 (bilateral)

### Parallel Mediation Analyses

**Fig. 1:**
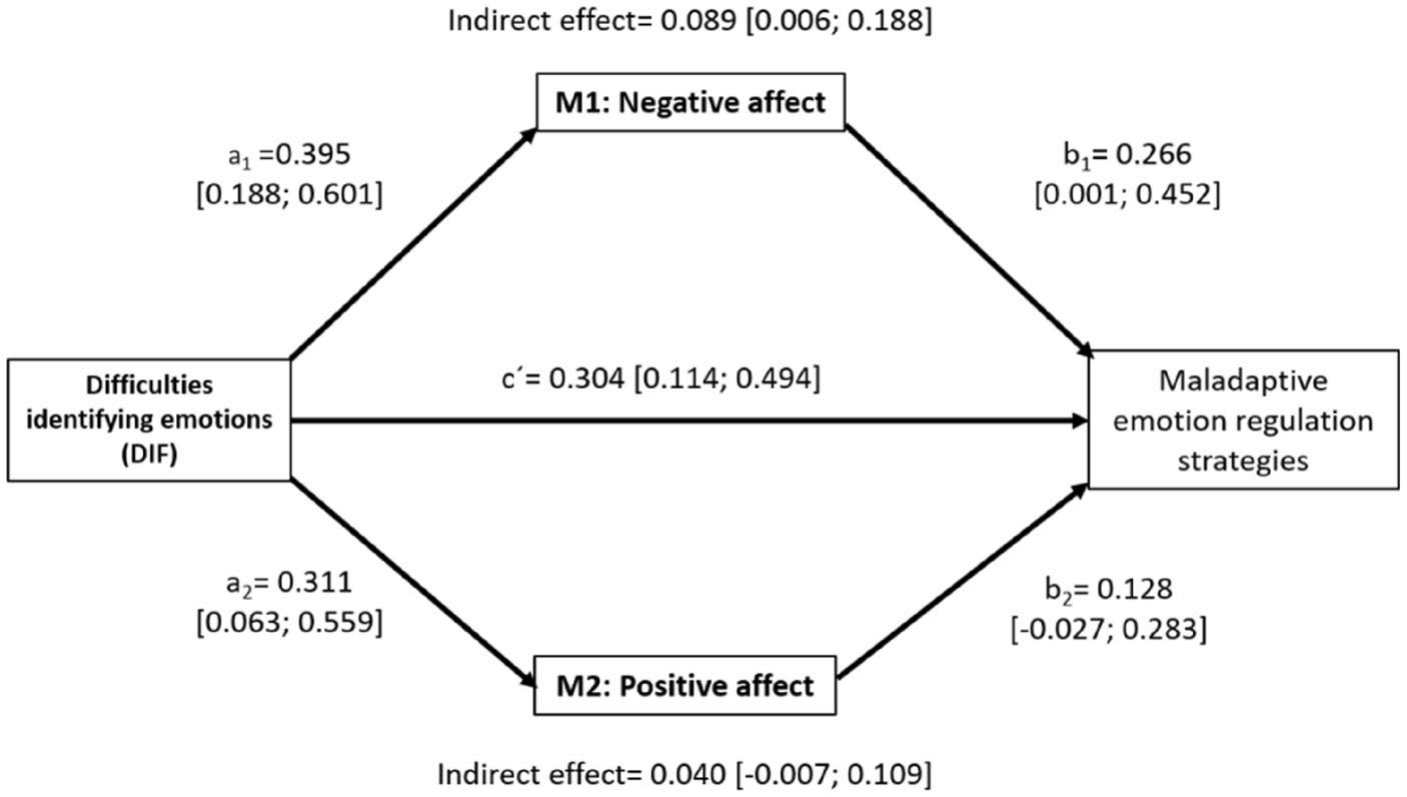
Parallel mediation analysis conducted in PROCESS v3.5 by entering two mediating variables. This mediation model corresponds to the 4th model in Preacher & Hayes (2008). *Note:* Both Beta coefficient values and 95% bootstrapping confident intervals are shown for each path

The results obtained for ARG are depicted in Fig. 1. Results of adolescents without problematic gambling are not depicted, as there were no statistically significant indirect effects in the mediation analysis.

Results from the parallel mediation analysis in the ARG group showed that greater difficulties to identify feelings (DIF) were indirectly related to higher use of MERS through its relationship with the Negative Affect subscale of the PANAS, but not with the Positive Affect subscale. First, as seen in Fig. 1, individuals with higher DIF showed higher Negative Affect (a_1_ = 0.395, CI [0.188; 0.601]) and higher Positive Affect (a_2_ = 0.311, CI [0.063; 0.559]). Secondly, higher Negative Affect was related to more use of MERS (b_1_ = 0.266, CI [0.001; 0.452]), whereas higher Positive Affect was not significantly related to MERS (b_2_ = 0.128, CI [-0.027; 0.283]). A 95% bias-corrected confidence interval based on 5000 samples indicated that the indirect effect through Negative Affect (DIF → Negative Affect → MERS; a_1_b_1_ = 0.089), holding Positive Affect constant, was significant [0.006; 0.188] as the confidence intervals did not include zero. Conversely, the indirect effect through Positive Affect (DIF → Positive affect → MERS; a_2_b_2_ = 0.040) was not significant (CI [-0.007; 0.109]). In other words, individuals with higher difficulties identifying feelings employ more MERS when they feel negative affect, but not when they feel positive affect. Furthermore, regarding the direct effect, higher DIF was associated with MERS when taking into account DIF´s indirect effect through the positive and negative dimensions of affect (c´ = 0.304, CI [0.114; 0.494]).

## Discussion

This study’s first aim was to analyze the differences in MERS, alexithymia, and negative and positive affect among adolescents balanced for gender and sex, with and without risky gambling behavior. According to the results, the differences were significant in negative affect and gambling severity. These results follow the line of those obtained by Källmén et al. (2008), who found that problem gamblers had a greater tendency to show depressive mood in comparison with non-gamblers. Bonnaire et al. (2017) also found higher levels of depression in pathological gamblers when compared with non-pathological gamblers, whereas Maniaci et al. (2017) found a greater tendency to show negative emotional states such as anger. Nevertheless, another study, conducted by Suen et al. (2017), found significant differences between non-problem and problem gamblers in variables such as impulsivity and risk-tolerance, whereas Jara-Rizzo et al. (2019) highlighted the potential relationship between affect-mediated impulsivity and gambling-related cognitions in gamblers. In the same line, other studies have found greater levels of alexithymia in university students with problem gambling behavior in comparison with non-problem gamblers (Parker et al., 2005). Moreover, individuals with gambling disorder use MERS more frequently to control negative emotional states than do individuals without gambling disorder (Estévez et al., 2020; Navas et al., 2016). The results of this study interesting because most previous studies do not analyze the specific differences among clinical and community samples (Jara-Rizzo et al., 2019; Källmén et al., 2008). Additionally, previous studies comparing clinical and community samples have presented the limitation of age range variability between problem and non-problem gamblers (Estevez et al., 2020). Clinical samples, on average, tend to be older because, despite early initiation of gambling behavior, even five years may go by until the behavior becomes problematic (López-Torres et al., 2020). Young people especially tend to need more time until they request treatment (Messerlian et al., 2005; Petry, 2002; Sharman et al., 2019). Moreover, several previous studies have samples composed mostly of men, whereas our study balanced the results by gender (McCarthy et al., 2018; Ronzitti et al., 2016). This difference is relevant, as women may show a different gambling profile from men, with higher levels of depression, negative affect, and a greater tendency to use gambling as a way of regulating emotions (Baggio et al., 2018; Carneiro et al., 2020; Merkouris et al., 2016). Nevertheless, most studies comprising women have included older women, who may start gambling after presenting emotional disorders such as depression, whereas our study included younger women, who may show a different profile from older women (Granero et al., 2018).

The second aim of the study was to analyze the relationship between the studied variables separately, as a function of the presence of problematic gambling behavior. Results showed that MERS were related to negative and positive affect in the ARG group, whereas positive affect was not related in the group without gambling problems. This agrees with previous studies in which gambling disorder was related to difficulties in emotion regulation, sensation-seeking, and negative emotional states such as depression and anxiety (Tarrega et al., 2015; Torrado et al., 2020). Regarding positive affect, the identification and regulation of positive affect may be relevant in problematic gambling behavior, as it has been suggested that difficulties to manage positive affect may involve difficulties to stop gambling behavior in individuals with problem gambling (Marchica et al., 2020; Rogier et al., 2020). Regarding negative affect, young people tend to gamble as a means to relieve negative emotions (Marchica et al., 2020). That is, gambling may be a dysfunctional way of regulating affect, increasing positive affect or suppressing negative affect (Hudson et al., 2013). Therefore, negative emotional states may favor the initiation and maintenance of problem gambling and are related to more stable and severe gambling behaviors (Atkinson et al., 2012; Wong et al., 2018) with more relapses (Daughters et al., 2005). However, other studies have suggested that not negative affect but the desire to gamble is what predicts gambling behavior (Quilty et al., 2017). In this line, it has been highlighted that adolescents’ difficulties in emotion regulation are related to difficulties to control their behavior when experiencing negative emotional states, and may thus promote gambling problems (Ciccarelli et al., 2020). Moreover, it has recently been found that individuals with a gambling disorder expressed less implicit emotions, such as anger and anxiety, and expressed more explicit emotions than non-gamblers (Guerrero-Vaca et al., 2020). They also showed a higher degree of anger and difficulties in emotional processing (Maniaci et al., 2017). The results of Maniaci et al. (2017) also highlight that difficulties in emotion identification were related to negative affect and MERS in both groups. These results agree with those of DiTrani et al. (2017), who suggested that the lack of identification and differentiation of inner emotional events may be related to problem gambling. Other studies also suggest that difficulties to stop gambling behavior may be related to alexithymia, which promotes loss-chasing behavior as a consequence of the incapacity to process the negative emotional consequences of such losses (Bibby, 2016). This author also suggested that gamblers with high levels of alexithymia may misinterpret or ignore the negative emotions associated with losses. Another result of our study is the relationship of alexithymia with MERS and negative affect. Previous studies have found that alexithymia may favor the use of dysfunctional emotion regulation as a way of increasing emotional arousal and avoiding negative emotions (Marchetti et al., 2019). Therefore, greater levels of alexithymia have been related to greater severity and intensity of gambling behavior (Bonnaire et al., 2017; Maniaci et al., 2017). Nevertheless, few previous studies have analyzed this relationship in samples of adolescents with and without problematic gambling.

Finally, the third aim of this study included the analysis of the mediating role of positive and negative affect in the relationship between alexithymia and MERS in the ARG group, finding that negative affect mediated the relationship between difficulties to identify emotions and the use of MERS in this group but not in the non-problematic gambling group. These results agree with previous studies that show that negative affect mediated between alexithymia and the increase of the severity of gambling disorder (Nöel et al., 2018), between gambling-related cognitions and gambling severity (Wong et al., 2018), and between reward-related responses and gambling severity (Atkinson et al., 2012). Moreover, these results verify the postulates of Rogier and Velotti (2018), who highlighted that identification of emotions is a relevant component that precedes the emotion regulation process, and therefore, emotional states that are difficult to manage may be implicated in the development, maintenance, and severity of gambling disorder. Furthermore, the need for recognizing one’s emotional states has been pointed out as necessary for effective, correct emotion-regulation processes (Weis et al., 2015). That is, emotional awareness and identification and understanding may be pre-requisites for the capacity to manage and regulate emotions adequately (Boden & Thompson, 2015). In this regard, a study conducted with adolescents showed that greater emotional awareness was associated with a better emotion-regulation process (Riley et al., 2019). In other behavioral addictions such as gaming addiction, previous studies also indicate that regular gamers obtain higher scores in alexithymic traits, as well as showing more difficulties to express emotions than irregular gamers (Gaetan et al., 2016). In the same way, higher scores in alexithymia have been related to emotional dysregulation, greater dependency severity, and a decreased capacity to remain abstinent in individuals with alcohol use disorder (Ghorbani et al., 2017; Stasiewicz et al., 2012). Therefore, these studies support the role of identification of emotions as a precursor of the use of MERS, which would be directly related to gambling behavior, and they verify previous theoretical postulates about the relationship between gambling disorder and emotion regulation (Ciccarelli et al., 2020; Jara-Rizzo et al., 2019; Schreiber et al., 2012; Williams et al., 2012). Moreover, these results were confirmed in an adolescent ARG group, in contrast with a non-problematic gambling group, which highlights the importance of these results for the understanding of gambling behavior in adolescents and young adults.

This study presents some limitations. First, it is a cross-sectional study, so causal relationships cannot be established. Additionally, SOGS-RA (Winters et al., 1993) is a screening instrument, so the results of this study might be different if clinical diagnoses were employed. Moreover, this study focuses on adolescents and young adults, so the results could be different in adult samples. Finally, this study focused on MERS based on previous theoretical and empirical postulates (Di Trani et al., 2017; Rogier & Velotti, 2018) but it would be interesting to conduct further studies analyzing more variables related to the presence of adaptive ERS that may be relevant in other gambling-related processes (Navas et al., 2016).

As a conclusion, this study highlights the importance of understanding alexithymia and emotion regulation processes in individuals with at-risk gambling, in which a relationship between difficulties to identify emotions, negative affect, and MERS was found. According to these results, the acquisition of adequate emotion identification skills, especially negative emotions, may be relevant to avoid the use of MERS that may promote problematic gambling behaviors. These results are of interest for clinical interventions, as they increase our comprehension of the processes underlying gambling behavior and specify which variables should be addressed. These results may also be of interest for developing prevention programs that focus on better emotion identification and management.

## References

[CR1] Atkinson J, Sharp C, Schmitz J, Yaroslavsky I (2012). Behavioral activation and inhibition, negative affect, and gambling severity in a sample of young adult college students. Journal of Gambling Studies.

[CR2] Bagby RM, Parker JD, Taylor GJ (1994). The twenty-item toronto alexithymia Scale—I. Item selection and cross-validation of the factor structure. Journal of Psychosomatic Research.

[CR3] Bagby RM, Taylor GJ, Parker JD (1994). The twenty-item toronto alexithymia scale—II. Convergent, discriminant, and concurrent validity. Journal of Psychosomatic Research.

[CR4] Baggio S, Gainsbury SM, Starcevic V, Richard JB, Beck F, Billieux J (2018). Gender differences in gambling preferences and problem gambling: A network-level analysis. International Gambling Studies.

[CR5] Bibby PA (2016). Loss-chasing, alexithymia, and impulsivity in a gambling task: Alexithymia as a precursor to loss-chasing behavior when gambling. Frontiers in Psychology.

[CR6] Billieux J, Lagrange G, Van der Linden M, Lançon C, Adida M, Jeanningros R (2012). Investigation of impulsivity in a sample of treatment-seeking pathological gamblers: A multidimensional perspective. Psychiatry Research.

[CR7] Blain B, Richard Gill P, Teese R (2015). Predicting problem gambling in Australian adults using a multifaceted model of impulsivity. International Gambling Studies.

[CR8] Boden MT, Thompson RJ (2015). Facets of emotional awareness and associations with emotion regulation and depression. Emotion.

[CR9] Bonnaire C, Barrault S, Aïte A, Cassotti M, Moutier S, Varescon I (2017). Relationship between pathological gambling, alexithymia, and gambling type. The American Journal on Addictions.

[CR10] Bradizza CM, Brown WC, Ruszczyk MU, Dermen KH, Lucke JF, Stasiewicz PR (2018). Difficulties in emotion regulation in treatment-seeking alcoholics with and without co-occurring mood and anxiety disorders. Addictive Behaviors.

[CR11] Brewer R, Cook R, Bird G (2016). Alexithymia: A general deficit of interoception. Royal Society Open Science.

[CR12] Calado F, Alexandre J, Griffiths MD (2017). Prevalence of adolescent problem gambling: A systematic review of recent research. Journal of Gambling Studies.

[CR13] Carneiro E, Tavares H, Sanches M, Pinsky I, Caetano R, Zaleski M, Laranjeira R (2020). Gender differences in gambling exposure and at-risk gambling behavior. Journal of Gambling Studies.

[CR14] Chester DS, Lynam DR, Milich R, Powell DK, Andersen AH, DeWall CN (2016). How do negative emotions impair self-control? A neural model of negative urgency. NeuroImage.

[CR15] Ciccarelli M, Nigro G, D’Olimpio F, Griffiths MD, Cosenza M (2020). Mentalizing failures, emotional dysregulation, and cognitive distortions among adolescent problem gamblers. Journal of Gambling Studies.

[CR16] Daughters SB, Lejuez CW, Strong DR, Brown RA, Breen RB, Lesieur HR (2005). The relationship among negative affect, distress tolerance, and length of gambling abstinence attempt. Journal of Gambling Studies.

[CR17] Di Trani M, Renzi A, Vari C, Zavattini GC, Solano L (2017). Gambling disorder and affect regulation: The role of alexithymia and attachment style. Journal of Gambling Studies.

[CR18] Domínguez-Sánchez FJ, Lasa-Aristu A, Amor PJ, Holgado-Tello FP (2013). Psychometric properties of the Spanish version of the Cognitive Emotion Regulation Questionnaire. Assessment.

[CR19] Elmas HG, Cesur G, Oral ET (2017). Alexithymia and pathological gambling: The mediating role of difficulties in emotion regulation. Turk Psikiyatri Dergisi.

[CR20] Estévez A, Herrero D, Sarabia I, Jauregui P (2014). El papel mediador de la regulación emocional entre el juego patológico, uso abusivo de internet y videojuegos y la sintomatología disfuncional en jóvenes y adolescentes. Adicciones.

[CR21] Estévez A, Jáuregui P, Lopez-Gonzalez H, Mena-Moreno T, Lozano-Madrid M, Macia L, Granero R, Mestre-Bach G, Steward T, Fernández-Aranda F, Gómez-Peña M, Moragas L, Del Pino-Gutierrez A, Codina E, Testa G, Vintró-Alcaraz C, Agüera Z, Munguía L, Baenas I, Valenciano-Mendoza E, Mora B, Menchón JM, Jiménez-Murcia S (2020). The severity of gambling and gambling related cognitions as predictors of emotional regulation and coping strategies in adolescents. Journal of Gambling Studies.

[CR22] Gaetan S, Bréjard V, Bonnet A (2016). Video games in adolescence and emotional functioning: Emotion regulation, emotion intensity, emotion expression, and alexithymia. Computers in Human Behavior.

[CR23] Garnefski N, Kraaij V, Spinhoven P (2001). Negative life events, cognitive emotion regulation and depression. Personality and Individual Differences.

[CR24] GarridoFernández M, Moral Arroyo GD, JaénRincón P (2017). Antecedentes de juego y evaluación del sistema familiar de una muestra de jóvenes jugadores patológicos [Assessment of gambling history and family system in a sample of adolescent pathological gamblers]. Health and Addictions/salud y Drogas.

[CR25] Ghorbani F, Khosravani V, Bastan FS, Ardakani RJ (2017). The alexithymia, emotion regulation, emotion regulation difficulties, positive and negative affects, and suicidal risk in alcohol-dependent outpatients. Psychiatry Research.

[CR26] González-Roz A, Fernández-Hermida JR, Weidberg S, Martínez-Loredo V, Secades-Villa R (2017). Prevalence of problem gambling among adolescents: A comparison across modes of access, gambling activities, and levels of severity. Journal of Gambling Studies.

[CR27] Granero R, Fernández-Aranda F, Mestre-Bach G, Steward T, García-Caro B, Prever F, Gavriel-Fried B, del Pino-Gutiérrez A, Moragas L, Aymamí N, Gómez-Peña M, Mena-Moreno T, Martín-Romera V, Menchón JM, Jiménez-Murcia S (2018). Clustering of treatment-seeking women with gambling disorder. Journal of Behavioral Addictions.

[CR28] Gross JJ (2002). Emotion regulation: Affective, cognitive, and social consequences. Psychophysiology.

[CR29] Guerrero-Vaca D, Granero R, Fernández-Aranda F, Mestre-Bach G, Martín-Romera V, Mallorquí-Bagué N, Aymamí N, del Pino-Gutiérrez A, Gómez-Peña M, Moragas L, Agüera Z, Vintró-Alcaraz C, Lozano-Madrid M, Menchón JM, Tárrega S, Munguía L, Jiménez-Murcia S (2020). Explicit and Implicit Emotional Expression in Gambling Disorder Measured by a Serious Game: A Pilot Study. Journal of Gambling Studies.

[CR30] Hudson A, Jacques S, Stewart SH (2013). Selective attention to emotional pictures as a function of gambling motives in problem and nonproblem gamblers. Psychology of Addictive Behaviors.

[CR31] Jara-Rizzo MF, Navas JF, Catena A, Perales JC (2019). Types of emotion regulation and their associations with gambling: A cross-sectional study with disordered and non-problem Ecuadorian gamblers. Journal of Gambling Studies.

[CR32] Jauregui P, Estevez A, Urbiola I (2016). Pathological gambling and associated drug and alcohol abuse, emotion regulation, and anxious-depressive symptomatology. Journal of Behavioral Addictions.

[CR33] Jiménez-Murcia S, Alvarez-Moya EM, Stinchfield R, Fernández-Aranda F, Granero R, Aymamí N, Gómez-Peña M, Jaurrieta N, Bove F, Menchón JM (2010). Age of onset in pathological gambling: Clinical, therapeutic and personality correlates. Journal of Gambling Studies.

[CR34] Johnson SL, Tharp JA, Peckham AD, Carver CS, Haase CM (2017). A path model of different forms of impulsivity with externalizing and internalizing psychopathology: Towards greater specificity. British Journal of Clinical Psychology.

[CR35] Källmén H, Andersson P, Andren A (2008). Are irrational beliefs and depressive mood more common among problem gamblers than non-gamblers? A survey study of Swedish problem gamblers and controls. Journal of Gambling Studies.

[CR36] Kandri TA, Bonotis KS, Floros GD, Zafiropoulou MM (2014). Alexithymia components in excessive internet users: A multi-factorial analysis. Psychiatry research.

[CR37] Kang K, Ok JS, Kim H, Lee KS (2019). The gambling factors related with the level of adolescent problem gambler. International Journal of Environmental Research and Public Health.

[CR38] Khantzian EJ (1985). The self-medication hypothesis of addictive disorders: Focus on heroin and cocaine dependence. American Journal of Psychiatry.

[CR39] Lamas, J. L., Santolaria, R., Estévez, A., & Jáuregui, P. (2018). *Guía clínica específica “jóvenes y juego online” [Specific Clinical Guide "young people and onlinegambling."]*. Funded by the Spanish Delegation of National Drugs Plan.

[CR40] Lesieur HR, Blume SB (1987). The South Oaks Gambling Screen (SOGS): A new instrument for the identification of pathological gamblers. American Journal of Psychiatry.

[CR41] López-Torres I, León-Quismondo L, Ibáñez A (2020). Actualización del perfil del jugador patológico [Updates on pathological gambler’s profiles]. RIECS.

[CR42] Lyvers M, Kohlsdorf SM, Edwards MS, Thorberg FA (2017). Alexithymia and mood: Recognition of emotion in self and others. American Journal of Psychology.

[CR43] Maniaci G, Picone F, van Holst RJ, Bolloni C, Scardina S, Cannizzaro C (2017). Alterations in the emotional regulation process in gambling addiction: The role of anger and alexithymia. Journal of Gambling Studies.

[CR44] Marchetti D, Verrocchio MC, Porcelli P (2019). Gambling problems and alexithymia: A systematic review. Brain Sciences.

[CR45] Marchica LA, Mills DJ, Derevensky JL, Montreuil TC (2019). The role of emotion regulation in video gaming and gambling disorder: A systematic review. Canadian Journal of Addiction.

[CR46] Marchica LA, Keough MT, Montreuil TC, Derevensky JL (2020). Emotion regulation interacts with gambling motives to predict problem gambling among emerging adults. Addictive Behaviors.

[CR47] Martínez-Sánchez F (1996). Adaptación española de la escala de Alexitimia de Toronto (TAS-20) [Spanish adaptation of the Toronto Alexithymia Scale TAS-20]. Clínica y Salud.

[CR48] McCarthy S, Thomas SL, Randle M, Bestman A, Pitt H, Cowlishaw S, Daube M (2018). Women’s gambling behaviour, product preferences, and perceptions of product harm: Differences by age and gambling risk status. Harm Reduction Journal.

[CR49] McRae K, Gross JJ (2020). Emotion regulation. Emotion.

[CR50] Merkouris SS, Thomas AC, Shandley KA, Rodda SN, Oldenhof E, Dowling NA (2016). An update on gender differences in the characteristics associated with problem gambling: A systematic review. Current Addiction Reports.

[CR51] Messerlian C, Derevensky J, Gupta R (2005). Youth gambling problems: A public health perspective. Health Promotion International.

[CR52] Morie KP, Yip SW, Nich C, Hunkele K, Carroll KM, Potenza MN (2016). Alexithymia and addiction: A review and preliminary data suggesting neurobiological links to reward/loss processing. Current Addiction Reports.

[CR53] Navas JF, Verdejo-Garcia A, Lopez-Gomez M, Maldonado A, Perales JC (2016). Gambling with rose-tinted glasses on: Use of emotion regulation strategies correlates with dysfunctional cognitions in gambling disorder patients. Journal of Behavioral Addictions.

[CR54] Noël X, Saeremans M, Kornreich C, Bechara A, Jaafari N, Fantini-Hauwel C (2018). On the processes underlying the relationship between alexithymia and gambling severity. Journal of Gambling Studies.

[CR55] Orlowski S, Bischof A, Besser B, Bischof G, Rumpf HJ (2019). Deficits in emotion regulation strategies among problematic and pathological gamblers in a sample of vocational school students. Journal of Behavioral Addictions.

[CR56] Parker JD, Wood LM, Bond BJ, Shaughnessy P (2005). Alexithymia in young adulthood: A risk factor for pathological gambling. Psychotherapy and Psychosomatics.

[CR57] Petry NM (2002). A comparison of young, middle-aged, and older adult treatment-seeking pathological gamblers. The Gerontologist.

[CR59] Quilty LC, Watson C, Toneatto T, Bagby RM (2017). A prospective investigation of affect, the desire to gamble, gambling motivations and gambling behavior in the mood disorders. Journal of Gambling Studies.

[CR60] Riley TN, Sullivan TN, Hinton TS, Kliewer W (2019). Longitudinal relations between emotional awareness and expression, emotion regulation, and peer victimization among urban adolescents. Journal of Adolescence.

[CR61] Rogier G, Velotti P (2018). Conceptualizing gambling disorder with the process model of emotion regulation. Journal of Behavioral Addictions.

[CR62] Rogier G, Moccia L, Di Nicola M, Velotti P (2018). Positive and negative urgency among addicted gamblers: The role of emotional suppression. International Conference on Behavioral Addictions.

[CR63] Rogier G, Colombi F, Velotti P (2020). A brief report on dysregulation of positive emotions and impulsivity: their roles in gambling disorder. Current Psychology.

[CR64] Ronzitti S, Lutri V, Smith N, Clerici M, Bowden-Jones H (2016). Gender differences in treatment-seeking British pathological gamblers. Journal of Behavioral Addictions.

[CR65] Ruiz de Lara CM, Navas JF, Soriano-Mas C, Sescousse G, Perales JC (2018). Regional grey matter volume correlates of gambling disorder, gambling-related cognitive distortions, and emotion-driven impulsivity. International Gambling Studies.

[CR66] Sancho, M., De Gracia De Gregorio, M., Granero, R., González-Simarro, S., Sánchez, I., Fernandez-Aranda, F., Trujols, J., Mallorquí-Bagué1, N., Mestre-Bach, G., del Pino-Gutiérrez, A., Mena-Moreno, T., Vintró-Alcaraz, C., Steward, T., Aymamí, N., Gómez-Peña, M., Menchón, J. M., & Jiménez-Murcia, S. (2019). Differences in emotion regulation considering sex, age and gambling preferences in a sample of gambling disorder patients. *Frontiers in Psychiatry, 10*, 1-9. doi: 10.3389/fpsyt.2019.00625. 10.3389/fpsyt.2019.00625 PMC6749049 31572231

[CR67] Sandín B, Chorot P, Lostao L, Joiner TE, Santed MA, Valiente RM (1999). Escalas PANAS de afecto positivo y negativo: Validación factorial y convergencia transcultural [PANAS scales of positive and negative affect: Factorial validation and cross-cultural convergence]. Psicothema.

[CR68] Schreiber LR, Grant JE, Odlaug BL (2012). Emotion regulation and impulsivity in young adults. Journal of Psychiatric Research.

[CR69] Secades-Villa, R., & Villa-Canal, A. (1998). *El juego patológico. Prevención, evaluación y tratamiento en la adolescencia* [Pathological gambling: Prevention, assessment, and treatment in adolescence]. Pirámide.

[CR70] Secades-Villa R, Calafat A, Fernández-Hermida JR, Juan M, Duch M, Skarstrand E, Becoña E, Talic S (2014). Duración del uso de internet y efectos psicosociales adversos entre los adolescentes europeos [Lenght of use of Internet and adverse psychosocial effects among European adolescents]. Adicciones.

[CR71] Secades-Villa R, Martínez-Loredo V, Grande-Gosende A, Fernández-Hermida JR (2016). The relationship between impulsivity and problem gambling in adolescence. Frontiers in Psychology.

[CR72] Sharman S, Murphy R, Turner J, Roberts A (2019). Psychosocial correlates in treatment seeking gamblers: Differences in early age onset gamblers vs later age onset gamblers. Addictive Behaviors.

[CR73] Shead NW, Callan MJ, Hodgins DC (2008). Probability discounting among gamblers: Differences across problem gambling severity and affect-regulation expectancies. Personality and Individual Differences.

[CR74] Sheppes G, Suri G, Gross JJ (2015). Emotion regulation and psychopathology. Annual Review of Clinical Psychology.

[CR75] Sideli L, La Barbera D, Montana S, Sartorio CR, Seminerio F, Corso M, Giunta S, Mannino G, La Cascia C (2018). Pathological gambling in adolescence: A narrative review. Mediterranean Journal of Clinical Psychology.

[CR76] Stasiewicz PR, Bradizza CM, Gudleski GD, Coffey SF, Schlauch RC, Bailey ST, Bole CW, Gulliver SB (2012). The relationship of alexithymia to emotional dysregulation within an alcohol dependent treatment sample. Addictive Behaviors.

[CR77] Suen V, Brown MR, Morck RK, Cribben I, Silverstone PH (2017). Risk tolerance, impulsivity, and self-esteem: Differences and similarities between gamblers and non-gamblers in a pilot study. Advances in Social Sciences Research Journal.

[CR78] Tárrega S, Castro-Carreras L, Fernández-Aranda F, Granero R, Giner-Bartolomé C, Aymamí N, Gómez-Peña M, Santamaría JJ, Forcano L, Steward T, Menchón JM, Jiménez-Murcia S (2015). A serious videogame as an additional therapy tool for training emotional regulation and impulsivity control in severe gambling disorder. Frontiers in Psychology.

[CR79] Terrone G, Musetti A, Raschielli S, Marino A, Costrini P, Mossi P, Salvatore S, Caretti V (2018). Attachment relationships and internalization and externalization problems in a group of adolescents with pathological gambling disorder. Clinical Neuropsychiatry.

[CR80] Torrado M, Bacelar-Nicolau L, Skryabin V, Teixeira M, Eusébio S, Ouakinin S (2020). Emotional dysregulation features and problem gambling in university students: A pilot study. Journal of Addictive Diseases.

[CR81] Volberg RA, Gupta R, Griffiths MD, Olason DT, Delfabbro P (2010). An international perspective on youth gambling prevalence studies. International Journal of Adolescent Medicine and Health.

[CR82] Watson D, Clark LA, Tellegen A (1988). Development and validation of brief measures of positive and negative affect: The PANAS scales. Journal Personality and Social Psychology.

[CR83] Weis N, Gratz K, Lavender J (2015). Factor structure and initial validation of a Multidimensional Measure of Difficulties in the Regulation of Positive Emotions: The DERS Positive. Behavior Modification.

[CR84] Williams AD, Grisham JR, Erskine A, Cassedy E (2012). Deficits in emotion regulation associated with pathological gambling. British Journal of Clinical Psychology.

[CR85] Winters KC, Stinchfield RD, Fulkerson J (1993). Toward the development of an adolescent gambling problem severity scale. Journal of Gambling Studies.

[CR86] Wong DFK, Zhuang XY, Jackson A, Dowling N, Lo HHM (2018). Negative mood states or dysfunctional cognitions: Their independent and interactional effects in influencing severity of gambling among chinese problem gamblers in Hong Kong. Journal of Gambling Studies.

